# Dysregulation of Neuronal Nicotinic Acetylcholine Receptor–Cholesterol Crosstalk in Autism Spectrum Disorder

**DOI:** 10.3389/fnmol.2021.744597

**Published:** 2021-10-11

**Authors:** Ana Sofía Vallés, Francisco J. Barrantes

**Affiliations:** ^1^Instituto de Investigaciones Bioquímicas de Bahía Blanca (UNS-CONICET), Buenos Aires, Argentina; ^2^Instituto de Investigaciones Biomédicas (BIOMED), UCA-CONICET, Buenos Aires, Argentina

**Keywords:** autism spectrum disorder, nicotinic receptor (nAChR), cholesterol, acetylcholine receptor-cholesterol interactions, perinatal period

## Abstract

Autism spectrum disorder (ASD) is a set of complex neurodevelopmental diseases that include impaired social interaction, delayed and disordered language, repetitive or stereotypic behavior, restricted range of interests, and altered sensory processing. The underlying causes of the core symptoms remain unclear, as are the factors that trigger their onset. Given the complexity and heterogeneity of the clinical phenotypes, a constellation of genetic, epigenetic, environmental, and immunological factors may be involved. The lack of appropriate biomarkers for the evaluation of neurodevelopmental disorders makes it difficult to assess the contribution of early alterations in neurochemical processes and neuroanatomical and neurodevelopmental factors to ASD. Abnormalities in the cholinergic system in various regions of the brain and cerebellum are observed in ASD, and recently altered cholesterol metabolism has been implicated at the initial stages of the disease. Given the multiple effects of the neutral lipid cholesterol on the paradigm rapid ligand-gated ion channel, the nicotinic acetylcholine receptor, we explore in this review the possibility that the dysregulation of nicotinic receptor-cholesterol crosstalk plays a role in some of the neurological alterations observed in ASD.

## Introduction

**Figure 1:**
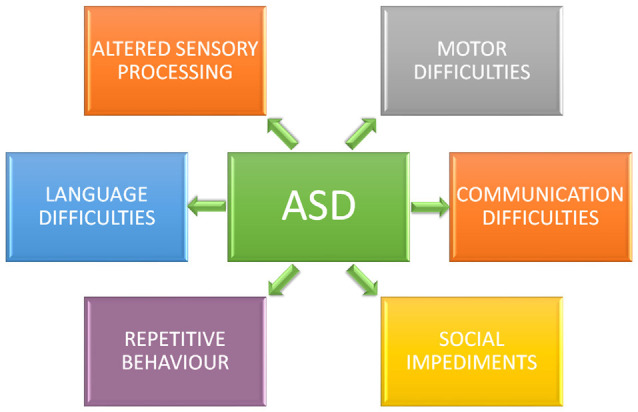
Core symptoms of autism spectrum disorder (ASD). Individuals diagnosticated with ASD present impaired social interaction, delayed and disordered language, repetitive or stereotypic behavior, restricted range of interests, and altered sensory processing.

Autism spectrum disorder (ASD) is a heterogeneous neurodevelopmental disorder manifested in childhood by deficits or atypicalities in both social behavior and cognitive function (Lai et al., 2014; Bonnet-Brilhault, 2017; Lord et al., 2018; Figure 1). About a third of children with ASD exhibit loss of skills or autistic regression (Tan et al., 2021). ASD onset symptoms are usually observed before three years of age. Very early symptoms -between 7 and 12 months- are reported in 41.9% of cases (Parmeggiani et al., 2019). Changes in social behavior or other subtle incipient autistic features may be noticed even during the first few months of life (Lord, 1995). These observations suggest that there are neuroanatomical and/or neurochemical alterations taking place early in the development of the central nervous system (CNS), and that such departure from normality is at the very root of the pathophysiology of the disorder.

**Figure:**
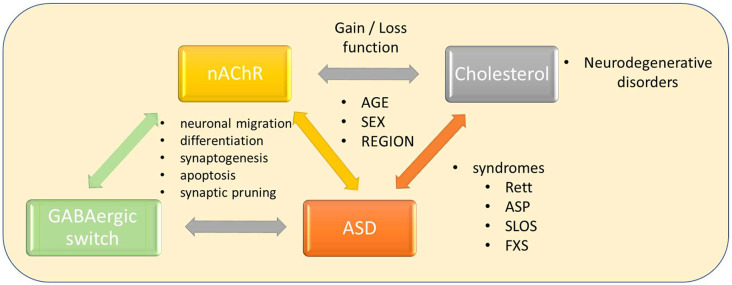
Interconnections between nicotinic and GABAergic neurotransmission, cholesterol, and neurodegenerative disorders, Autism Spectrum Disorder, and other syndromes.

ASD shows comorbidity with other neurological and neuropsychiatric disorders including epilepsy, developmental coordination disorder, substance abuse, sleep, mood, and immunological disorders (Marotta et al., 2020). In addition, there are several genetic syndromes commonly associated with autism, e.g., fragile X syndrome or Rett syndrome (Ornoy et al., 2016). All of these factors reflect a complex multifactorial etiopathology of ASD.

Neurotransmitters and neuropeptides are key players in normal brain development. Their fine-tuning is essential to neuronal cell migration, differentiation, synaptogenesis, apoptosis, and synaptic pruning (Quaak et al., 2013). Alteration of neurotransmitter systems may therefore lead to impairment of the complex dynamics of brain development. Data obtained from postmortem brains, molecular imaging, and genetic data have provided strong evidence of the involvement of dysfunctional neurotransmission in autism.

Recent studies have shown that ASD is associated with disruption of cholesterol metabolism (Wang, 2014; Cartocci et al., 2018; Segatto et al., 2019). The brain is the organ with the highest concentration of cholesterol, accounting for approximately 23% of the body’s cholesterol content, mainly due to the high membrane/volume ratio of neurons (Dietschy and Turley, 2004). Cholesterol deeply affects the properties of the plasma membrane where neurotransmitter receptors reside (Fantini and Barrantes, 2009). Cholesterol concentration alters the physical properties of the bulk membrane and promotes the formation of liquid-ordered (Lo) domains (van Meer et al., 2008). These domains display unique biophysical properties: they have a highly dynamic transient nature and differ structurally from the rest of the bulk lipid bilayer owing to their higher concentration in rigid, saturated acyl chains, cholesterol, and sphingolipids (Borroni et al., 2016), making these Lo domains thicker than the rest of the membrane. Such characteristics provide these domains with the ability to spatially and temporally confine signaling processes. In addition, cholesterol interacts with neurotransmitter receptors through consensus linear binding sequences like the so-called cholesterol recognition/interaction amino acid consensus motifs (CRAC and its mirror image CARC; Baier et al., 2011). These motifs have been proposed to facilitate membrane protein incorporation into cholesterol-rich domains in a great variety of membrane proteins, including the superfamily of pentameric ligand-gated ion channels (pLGIC) and the superfamily of G-protein coupled receptors (GPCR). The prototypic pLGIC, the nAChR, for instance, exhibits a CRAC motif adjacent to the transmembrane helix M1, and a CARC sequence on the M4-facing surface of M1 adjacent to one of the proposed cholesterol-binding cavities (Baier et al., 2011; Fantini et al., 2016). In addition, gain or loss of function of ion channels after acute cholesterol depletion or enrichment has been reported for the nAChR (Santiago et al., 2001; Borroni et al., 2007), the NMDA receptor (Antonini et al., 2018), the glycine receptor (Yao et al., 2020), and the serotonin 1A (Singh et al., 2007) and serotonin subtype-7 receptors (Sjögren et al., 2006).

During brain development, cholesterol plays a crucial role in synaptogenesis, membrane trafficking, signal transduction, and maturation of the CNS (Orth and Bellosta, 2012). Moreover, brain cells must regulate their cholesterol levels independently of the rest of the body, since the blood-brain barrier (BBB) prevents the entry of lipoproteins into the brain parenchyma (Dietschy and Turley, 2004). It is therefore not surprising that altered cholesterol homeostasis can perturb CNS development and function (Linetti et al., 2010; Segatto et al., 2014; Wang, 2014; Cartocci et al., 2016) and thus be implicated in the pathophysiology of ASD. Accordingly, many studies have reported that patients with ASD present perturbation in cholesterol metabolism (Schengrund et al., 2012; Wang, 2014; Gillberg et al., 2017; Petrov et al., 2017). In addition, the Rett, Asperger, and Smith-Lemli-Opitz syndromes associated with ASD are neurological disorders characterized by disruption of brain cholesterol homeostasis (Dziobek et al., 2007; Wang, 2014; Gillberg et al., 2017).

Cholinergic neurotransmission participates in the regulation of cognitive function (Bentley et al., 2011), including attention, memory and learning processes (Maurer and Williams, 2017), cognitive flexibility (Prado et al., 2017), and social interaction (Wang et al., 2015). Brain cholinergic system dysfunction has also been reported in ASD (Hardan and Handen, 2002; Eissa et al., 2018). Since nAChR function is influenced by its lipid microenvironment (see reviews in Barrantes, 2004, 2017) and cell-surface trafficking of nAChRs is dependent on cholesterol metabolism (Pediconi et al., 2004; Borroni et al., 2020) we explore in this review the various neurological alterations described in ASD and their possible association with an abnormal crosstalk between nAChRs and cholesterol.

## Cholesterol Metabolism in Brain

The importance of this lipid in the CNS is two-fold: (1) neurons require the sterol for normal functioning; and (2) cholesterol is an essential constituent of myelin sheaths formed by oligodendrocytes to insulate axons and plasma membranes of astrocytes and neurons (Fracassi et al., 2019). Cholesterol is also needed for synaptic vesicle formation and release (Pfrieger, 2003). The cholesterol-rich domains described in the Introduction have also been reported to occur in many types of synapses, and functional roles have been attributed to these specialized membrane domains (Suzuki, 2002; Gil et al., 2006; Allen et al., 2007; Wasser and Kavalali, 2009; Mailman et al., 2011).

**Figure 2:**
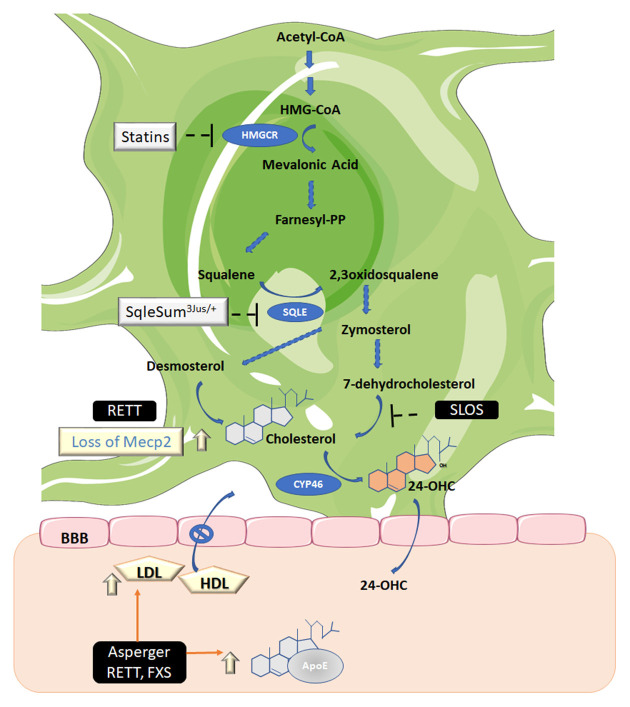
Biosynthetic and catabolic pathways in the brain. Cholesterol is the end product of the mevalonate pathway. Three molecules of acetyl-coenzyme A (acetyl-CoA) are required to generate 3-hydroxy-3-methyl-glutaryl CoA (HMG-CoA) which is the substrate of HMG-CoA reductase (HMGCR), the mevalonic acid-generating enzyme. This is the only limiting rate reaction in cholesterol synthesis and is the target of statins, the cholesterol-lowering drugs. Mevalonic acid is then converted to farnesyl diphosphate (farnesyl-PP) and squalene. The latter is then converted to 2,3 oxidosqualene by squalene epoxidase (SQLE). Then a series of enzymes catalyzes the synthesis of zymosterol, which can, in turn, be converted to cholesterol *via* desmosterol by 24-dehydrocholesterol reductase, also known as the Bloch pathway (operating in astrocytes) or *via* lathosterol (not shown), which is the precursor of 7-dehydrocholesterol, also known as the Kandutsch-Russell pathway (the minority pathway in neurons). The enzyme 7-dehydrocholesterol reductase, which converts 7-dehydrocholesterol to cholesterol, is defective in patients with SLOS. Cholesterol, in turn, must be converted to 24-*S*-hydroxycholesterol (24-OHC) by 24-hydroxylase (CYP46) to be able to permeate the blood-brain barrier (BBB). Asperger syndrome (Asperger); Smith–Lemli–Opitz syndrome (SLOS); Rett syndrome (RETT); Fragile-X syndrome (FXS); Apolipoprotein E (ApoE); methyl CpG binding protein 2 (Mecp2); low-density lipoproteins (LDL); high-density lipoproteins (HDL). Figure created using Servier Medical Art Commons Attribution 3.0 Unported License (http://smart.servier.com). Servier Medical Art by Servier is licensed under a Creative Commons Attribution 3.0 Unported License.

Cholesterol present in the CNS must be synthesized *in situ* (Jeske and Dietschy, 1980) since lipoproteins containing cholesterol are unable to cross the BBB. Cholesterol is synthesized from acetyl-CoA following the mevalonate pathway (Fracassi et al., 2019; Figure 2). The endoplasmic reticulum-resident protein 3-hydroxy-3-methylglutaryl coenzyme-A reductase (HMGCR), reduces 3-hydroxy-3-methylglutaryl coenzyme A (HMG-CoA) to mevalonic acid in the key rate-limiting and irreversible step of cholesterol biosynthesis. Brain cholesterol metabolism displays regional, age, and sex-specific differences. Experiments performed on different brain regions of adult male rats displayed very low HMGCR activity in the brain stem, whereas the hippocampus, brain cortex, and cerebellum showed high activity of this enzyme (Segatto et al., 2011, 2012; Fracassi et al., 2019; Figure 2).

During prenatal life neurons produce cholesterol, but after birth neurons cease to do so under normal conditions. The provision of cholesterol in the brain thus relies on its production by astrocytes (Mauch et al., 2001; Fracassi et al., 2019). If cholesterol biosynthesis exceeds the requisite levels, excess cholesterol is secreted to plasma. However, cholesterol must be first transformed into 24-*S* hydroxycholesterol (24-OHC) with the intervention of another important enzyme in cholesterol metabolism, 24-hydroxylase (CYP46; Figure 2). 24-OHC is able to cross the BBB. CYP46 accounts for approximately 40% of brain cholesterol conversion. It exists in specific brain areas, suggesting that neurons in some brain regions may be more sensitive to cholesterol levels than in others (Lund et al., 2003; Ramirez et al., 2008).

In addition to this main mechanism of cholesterol removal from the CNS (Leoni and Caccia, 2013; Boussicault et al., 2016), other cholesterol metabolites, such as 5α-hydroxy-6-oxocholesterol (3β,5α-dihydroxycholestan-6-one), 7β-hydroxycholesterol, and 7-oxocholesterol, generally considered to be formed through reactive oxygen species, are similarly exported from the brain (Iuliano et al., 2015).

## Cholesterol-Related Syndromes and ASD

The complex constellation of metabolic mechanisms discussed in the preceding section is subject to dysregulation, as is observed in various chronic neurodegenerative disorders (Shobab et al., 2005; Bi and Liao, 2010; Vancampfort et al., 2013; Leoni and Caccia, 2015; Jiang et al., 2020). In addition, there are various ASD-associated syndromes that also present alterations in cholesterol homeostasis. In this section, we briefly describe these pathologies.

### Rett Syndrome

Rett syndrome is caused by mutations in the gene encoding for the transcriptional regulator methyl CpG binding protein 2 (MECP2; Baker et al., 2013; Lyst et al., 2013; Xu and Pozzo-Miller, 2013). Mecp2 was the first autism spectrum disorder gene to be identified at the molecular level (Amir et al., 1999) and MECCP2 mutant mice showed disruption of cholesterol homeostasis (Buchovecky et al., 2013) consisting of a transient increase in brain cholesterol concentrations.

Either genetic (e.g., SqleSum^3Jus/+^) or pharmacological (e.g., statin-mediated) inhibition of the mevalonate-cholesterol pathway were shown to ameliorate behavioral and clinical symptoms. Loss of MECP2 disrupts cholesterol homeostasis and suggested that abnormal cholesterol metabolism may account for the pathogenesis of Rett (Buchovecky et al., 2013; Nagy and Ackerman, 2013), thus linking the autism-associated gene to cholesterol metabolism and providing further insight into the relationship between cholesterol metabolism and ASDs.

### Asperger Syndrome

Asperger syndrome is a neurodevelopmental disorder belonging to the ASD palette that is characterized by impairments in socialization and ritualistic and stereotypic behaviors. Genetic and early developmental factors are considered key factors. Increased total cholesterol and LDL-C levels have been observed in Asperger syndrome (Dziobek et al., 2007). Additionally, higher triglyceride levels, lower HDL, and higher (LDL)/(HDL) ratios were recorded in male children with ASD compared to healthy control individuals (Kim et al., 2010).

### Smith-Lemli-Opitz-Syndrome (SLOS)

A decline in cholesterol synthesis is observed in SLOS patients, who have a deficiency in the final step of the cholesterol biosynthetic pathway (Figure 2). This defect causes low or lower than normal plasma cholesterol levels and increased 7- and 8-dehydrocholesterol levels (Steiner et al., 2000; Svoboda et al., 2012). In parallel, ASD symptoms are frequently found in individuals with SLOS, along with intellectual disability, facial abnormalities, seizures, and other pathologies (Sikora et al., 2006; Aneja and Tierney, 2008). Cholesterol supplementation results in only slight improvements in SLOS symptoms and does not appear to be an effective treatment for SLOS. The lack of demonstrable effects may be due to the fact that dietary cholesterol, according to currently accepted evidence, does not cross the BBB (Jira et al., 2000; Björkhem and Meaney, 2004). Thus, more randomized controlled trials with standardized cholesterol dosing, laboratory monitoring of sterol levels, and well-defined outcome measures are required for a more objective assessment of the potential role of cholesterol as an effective palliative treatment for SLOS patients.

### Fragile X Syndrome

Fragile X syndrome (FXS) is associated with intellectual disability, behavioral dysfunction, and autistic features (Berry-Kravis et al., 2015). These authors found low HDL, low LDL and total cholesterol levels in all FXS patients at their institution. Later, Çaku and coworkers also found the presence of hypocholesterolemia in a French Canadian-FXS cohort, a condition that appeared to influence their clinical phenotype (Çaku et al., 2017). Although these studies show that peripheral cholesterol metabolism could be affected in FXS, further studies are required to explore whether this is the case.

## Apolipoproteins and ASD

Since brain cholesterol is synthesized locally, the cholesterol-carrying proteins, apolipoproteins (APO), are especially important for recycling brain cholesterol and for maintaining brain homeostasis (Björkhem and Meaney, 2004). Previous research has shown that these proteins are dysregulated in ASD. APO B-100 and APO A-IV have been reported to be augmented in children with high- vs. low-functioning autism (Corbett et al., 2007). APOA1 is present in neurons in the CNS and is a critical component of cholesterol biosynthesis/metabolism (Harr et al., 1996; Fujii et al., 2002).

ApoE in its various isoforms E2, E3, and E4, coded by the gene alleles ε2, ε3, and ε4, are a family of apolipoproteins also present in the CNS; the brain is the organ with the second-highest ApoE expression after the liver (Linton et al., 1991; Serrano et al., 2021). The ApoE isoforms differ from each other in only one amino acid residue. ApoE has a myriad of functions in the CNS, participating in lipid transport and metabolism, growth, maintenance, and repair of axons and myelin during neuronal development (see recent review in ref. Serrano et al., 2021). The major function of ApoE is its participation in cholesterol homeostasis, which is unique in the brain. ApoE is produced by astrocytes and to a lesser extent by oligodendrocytes, microglia, and ependymal layer cells (Mahley et al., 2006). While under normal conditions neurons do not express ApoE, they do so under certain conditions such as excitotoxic injury (Xu et al., 1999).

The association of ApoE variants with ASD is still a controversial issue. Distortions in the transmission of the *Apo*ε4 over *Apo*ε3 and *Apo*ε4 allele in families with ASD have been described (Persico et al., 2004). The ApoE2 protein variant displays the lowest receptor binding affinity for low-density lipoprotein (LDL) receptors compared with ApoE3 and ApoE4 (Weisgraber et al., 1982). In addition, ApoE2 and ApoE4 isoforms have been suggested to be involved in the complex etiological predisposition for ASD (Giunco et al., 2009). However, other authors have not found any association between ApoE and autism (Raiford et al., 2004; Ashley-Koch et al., 2007). More recently, abnormal ApoE methylation has been shown to be significantly associated with ASD (Lane-Donovan et al., 2016). The authors implied that ApoE hypermethylation may be regarded as a potential biomarker in ASD diagnosis. They further speculated that ApoE hypermethylation may reduce ApoE expression, eventually leading to the onset of ASD. Abnormal ApoE methylation and low levels of ApoE-particles in the brain correlate with an increased risk of Alzheimer disease (AD), which may have overlapping mechanisms with ASD. Interestingly, an ApoE knock-out mouse that fails to express ApoE in the brain but has a normal amount in the periphery, showed faltering synapses (Lane-Donovan et al., 2016). These data highlight that cholesterol homeostasis is crucial for neurons in the developing mammalian brain. Thus, dysregulation of either the level of these proteins or of cholesterol metabolism may contribute to the onset of ASD and serve as biomarkers for certain ASD subtypes.

## nAChRs and Neural Excitability

**Figure 3:**
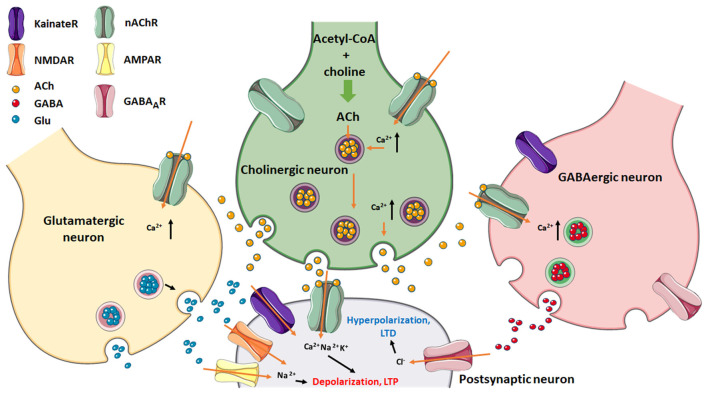
Postsynaptic neuron regulation *via* excitatory (glutamatergic/cholinergic) and inhibitory (GABAergic) afferents. Note the presence of nAChRs in the three presynaptic nerve endings as well as in the postsynaptic neuron. Glu can stimulate either NMDA- or AMPA-type glutamatergic receptors, which together with nAChRs, cause depolarization which leads to LTP of the postsynaptic membrane. GABA channel activation results in hyperpolarization of this membrane and favors LTD. Figure created using Servier Medical Art Commons Attribution 3.0 Unported License (http://smart.servier.com). Servier Medical Art by Servier is licensed under a Creative Commons Attribution 3.0 Unported License. Glu, glutamate.

Cholinergic signaling modulates the excitatory–inhibitory balance in the brain. Presynaptically or postsynaptically located nAChRs can influence synaptic plasticity by increasing intracellular Ca^2+^ release, induction of long-term potentiation (LTP), and favor a depolarization state (Figure 3).

nAChRs are excitatory cationic LGICs found in pre-, post-, and extra-synaptic membranes throughout the central and peripheral nervous system (Jones and Yakel, 1997; Wonnacott, 1997; Jones et al., 1999; Gotti et al., 2006; Colombo et al., 2013). The main role of nAChRs is neurotransmission across different types of synapses, where they mediate inter-neuronal communication and muscle contraction. There are 17 different subunits (α1–α10, β1–β4, γ, ε, and δ) encoded by 17 genes in vertebrates that combined, form either homo- or hetero-pentameric structures (Jones and Sattelle, 2010). The muscle-type nAChR found at the neuromuscular junction is formed from four distinct subunit types organized in an (α)_2_βγδ pentamer. In the adult, the fetal γ-subunit is replaced by the ε-subunit. In the human brain, both heteromeric α4β2 and homomeric α7 nAChRs are the most abundant while combinations such as α3β4, α3β2, and α6β2β3 nAChRs, etc., are less abundant and are only expressed in specific brain regions (Dani and Bertrand, 2007; Taly et al., 2009). The neuronal nAChRs are commonly found in the basal forebrain, hippocampus, cerebellum, and temporal cortex, brain locations considered to be involved in learning, cognition, and memory (Dineley et al., 2015). In the former location, activation of nAChRs results in the release of neurotransmitters including dopamine, norepinephrine, γ-aminobutyric acid (GABA), and glutamate (Glu) in a Ca^2+^-dependent manner. For example, activation of presynaptic α4β2 or α7 nAChRs depolarizes hippocampal interneurons, indirectly affecting neurotransmitters release (Glu or GABA) by activating voltage-gated calcium channels (McQuiston, 2014). Similarly, stimulation of postsynaptic nAChRs induces significant inward currents in neurons in many brain regions. In terms of synaptic plasticity, presynaptic α4β2 nAChR activation by nicotine has been reported to induce dendritic spine enlargement (Oda et al., 2014) by increasing the concentration of Glu and the activity of glutamatergic neurotransmission. Presynaptic α3β4 nAChR activation in parvalbumin-positive cells stimulates tetrodotoxin-insensitive GABA release *via* T-type voltage-gated calcium channels and Ca^2+^ from internal stores (Tang et al., 2011). α7 nAChR-mediated intracellular calcium signaling at synapses on hippocampal CA1 neurons can result in LTP after increased glutamate release (Ji et al., 2001). Postsynaptic glutamate receptors can also be regulated by α7 nAChR signaling at somatodendritic synapses (Alkondon et al., 1998; Duan et al., 2015) and thus modulate synaptic plasticity and GABAergic interneuron activity (Gu and Yakel, 2011; Pidoplichko et al., 2013). Interestingly, α7 nAChRs in the prelimbic cortex have a bidirectional role in modulating network excitability since both activation and inhibition of α7 nAChRs result in the induction of LTP (Udakis et al., 2016). These studies collectively help to understand the role of cholinergic receptors in neural excitability and plasticity.

Cholesterol levels have modulatory effects on nAChR function: acute cholesterol depletion results in a transient ion channel gain-of-function in the cell line CHO-K1/A5 cells and in *Torpedo* nAChR expressed in *Xenopus* oocytes, whereas cholesterol enrichment leads to loss-of-function (Santiago et al., 2001; Borroni et al., 2007). Thus, variations in brain cholesterol levels may impact the regulation of the polarization state of neuronal membranes and therefore have different outcomes in neurodevelopment.

### Activation of nAChRs in GABAergic Neurons

**Figure 4:**
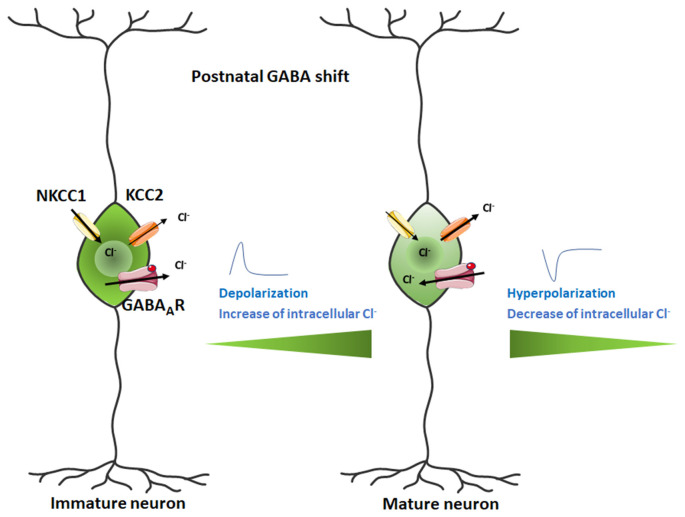
Switch in GABA action from excitatory in the developing mammalian brain to inhibitory in the postnatal brain. The Cl^−^ ion flux is reversed during the neurodevelopmental change, transforming the depolarizing current into a hyperpolarizing signal. Figure created using Servier Medical Art Commons Attribution 3.0 Unported License (http://smart.servier.com). Servier Medical Art by Servier is licensed under a Creative Commons Attribution 3.0 Unported License.

Activation of nAChRs in GABAergic neurons promotes GABA release whereas their inhibition prevents their release. During prenatal and early postnatal development GABA behaves as an excitatory neurotransmitter because of the relatively high expression of the Na^2+^/K^+^/Cl^−^ co-transporter 1 (NKCC1) and low expression of the K^+^/Cl^−^ co-transporter 2 (KCC2), thus setting the transmembrane Cl^−^ gradient in the brain and favoring a high internal Cl^−^ cellular concentration (Rivera et al., 1999; Ben-Ari, 2002; Payne et al., 2003; Shaw et al., 2020). As postnatal development proceeds, NKCC1 is downregulated and KCC2 is upregulated, resulting in an outflow of Cl^−^ from inside the cell and a switch to inhibitory GABA signaling. nAChR activation not only increases GABA release during development (Maggi et al., 2001) but can also regulate the switch from depolarizing to hyperpolarizing GABA_A_R-mediated signaling by controlling the levels of NKCC1 and KCC2 and therefore modulating chloride homeostasis (Liu et al., 2006). The postnatal shift from depolarizing to hyperpolarizing GABA responses is a crucial event in brain development (Figure 4). Alterations in its timing may account for some of the behavioral deficits observed in patients with ASD (Pizzarelli and Cherubini, 2011; Schulte et al., 2018). The timing of the GABA shift is strongly dependent on cell type, sex, and brain region (Peerboom and Wierenga, 2021). Chronic nicotine treatment during the first postnatal week increases excitatory GABAergic signaling in males to a greater extent than in females (Damborsky and Winzer-Serhan, 2012). This sex-specific susceptibility may be related to the fact that the GABAergic system matures earlier in females than in males (Nuñez and McCarthy, 2007; Galanopoulou, 2008). It is therefore highly probable that a variety of conditions impact GABAergic transmission leading to sex-specific developmental outcomes. The latter may thus contribute to understanding why males are disproportionally affected by neurodevelopmental disorders such as schizophrenia spectrum disorders and ASD (Bale et al., 2010).

Dysregulation of the GABAergic signaling could also alter the expression of brain-derived neurotrophic factor (BDNF) expression, which is increased by excitatory but not inhibitory GABA transmission (Berninger et al., 1995; Represa and Ben-Ari, 2005). In addition, lower levels of BDNF have been reported in MeCP2-mutant mice whereas overexpression of BDNF rescues several cellular and behavioral deficits in these mice (Chang et al., 2006; Pozzo-Miller et al., 2015).

The restoration of inhibitory GABA signaling in animal models of FXS and Rett syndrome during a restricted postnatal period has been shown to bring about significant improvements in brain function (Banerjee et al., 2016; He et al., 2019). There is growing evidence that the incidence of ASD is twice as high in moderate- to-late pre-term born children (Shaw et al., 2020). Furthermore, these pre-term children have a 33% higher chance of developing depression and a 58% higher probability of developing anxiety in childhood and adolescence (Schendel and Bhasin, 2008; Singh et al., 2013; Shaw et al., 2020).

Liu and coworkers show that when activation of nAChR is inhibited by antagonist administration, GABA induces depolarization on acutely dissociated chick ciliary ganglion neurons (Liu et al., 2006). Furthermore, α7 nAChR knockout also prevents the GABA switch and in mutant mice that carry the β2^Val287Leu^ mutant, the expression of KCC2 is lower in the prefrontal cortex than in controls, thus delaying the GABAergic shift (Amadeo et al., 2018). Dendritic morphological maturation during early development strongly depends on the excitatory action of GABA (Cancedda et al., 2007). Reducing the expression of NKCC1 in granule cells in the adult hippocampus prevents dendritic development (Ge et al., 2006). In agreement with this observation, premature KCC2 overexpression on neurons of the somatosensory cortex exhibit fewer and shorter dendritic processes (Cancedda et al., 2007).

To sum up, nAChRs regulate chloride homeostasis by modulating the switch from depolarizing to hyperpolarizing GABA_A_R-mediated signaling. Different levels of expression or function of nAChR in the prenatal period as a consequence of brain cholesterol variations (see “nAChR and Cholesterol” section), mutations, or nicotine exposure, may lead to an adverse physiopathological outcome considering the important role of this event for brain development.

### Activation of nAChRs in Glutamatergic Neurons

Cholinergic activation also modulates Glu release from glutamatergic presynaptic neurons thus contributing to postsynaptic activation of glutamate receptors. Glu modulation in the perinatal period is also of critical importance for normal neurodevelopment to proceed. Expression of several nAChR subunits that intervene in regulating synaptic maturation in the neocortex peaks between the 2nd and the 3rd postnatal week in mice. In particular, α7 nAChRs are thought to regulate dendritogenesis and the maturation of glutamatergic synapses (Campbell et al., 2010; Lozada et al., 2012a; Morley and Mervis, 2013; Lin et al., 2014). Likewise, nAChR containing β2 subunits participate in the formation of dendritic spines and regulate dendritic morphology (Ballesteros-Yáñez et al., 2010; Bailey et al., 2012; Lozada et al., 2012b; Kang et al., 2015). Thus, hyperfunction of nAChRs that alter Ca^2+^ permeability may indirectly modify GluR distribution in excitatory synapses through the modulation of the actin cytoskeleton that shapes spine structure (Lozada et al., 2012a,b).

Glutamatergic activity has also been associated with the upregulation of α7 nAChRs *via* BDNF, as observed in the hippocampus and other brain regions (Zhou et al., 2004). BDNF increases both cell-surface and intracellular α7 nAChR pools in dissociated rat hippocampal neurons (Vallés and Barrantes, 2012). This upregulation of α7 nAChR subunits depends on the glutamatergic activity. Since long exposure to BDNF is required to detect the increment of α7 nAChRs, Massey and coworkers suggested that de novo synthesis of the receptor occurs (Massey et al., 2006). It should be noted that the modulation of α7 nAChRs promotes cell depolarization through calcium entry into the postsynaptic cell, which in turn leads to a series of calcium-dependent events (Berg and Conroy, 2002) including changes in gene expression (Massey et al., 2006). In addition, α7 nAChRs can modulate the excitatory system *via* non-neuronal cells (Wang et al., 2013). Activation of α7 nAChRs in astrocytes promotes the development of glutamatergic networks by recruiting AMPA receptors to post-synaptic sites, thus suggesting an important role for cholinergic signaling in the conversion of ‘silent’ glutamatergic synapses into functional ones (Wang et al., 2013). Thus alteration of cholinergic transmission impacts glutamatergic transmission, affecting neuronal excitability, dendritrogenesis, intracellular signaling, and gene expression.

### Direct Modulation of GABAergic and Glutamatergic Receptors by Cholesterol

Changes in cholesterol can also directly impact GABA and glutamate receptor modulation. GABA_A_Rs are positively modulated and even activated by neurosteroids, endogenous cholesterol derivatives (Hénin et al., 2014). Two examples of potent, naturally occurring GABA_A_-active steroids are 3*α*-hydroxy-5*α*-pregnan-20-one (allopregnanolone) and 5*α*-pregnane-3*α*, 21-diol-20-one. Sooksawate and Simmonds reported the active modulation of the GABA_A_R by cholesterol (Sooksawate and Simmonds, 2001).

**Figure 5:**
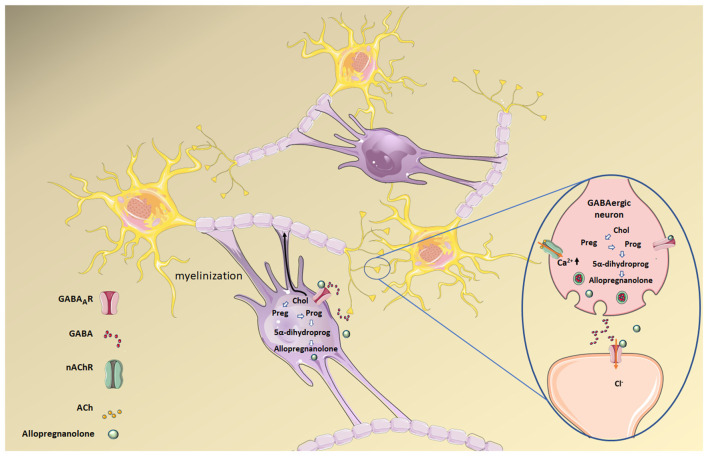
Allopregnanolone synthesis from cholesterol (Chol) in both mature oligodendrocytes (the cells responsible for forming the central myelin) and neurons. Allopregnanolone and other neurosteroids act as transcriptional factors that regulate gene expression upon back-oxidation into 5α-pregnane steroids, and also modulate neuronal excitability by interacting with neurotransmitter receptors. Allopregnanolone in particular is a positive allosteric modulator of the GABA_A_R. Preg, pregnenolone; Prog, progesterone; 5α-dihydroprog, 5α-dihydroprogesterone. Figure created using Servier Medical Art Commons Attribution 3.0 Unported License (http://smart.servier.com). Servier Medical Art by Servier is licensed under a Creative Commons Attribution 3.0 Unported License.

Allopregnanolone is mainly synthesized in the placenta and has an important role during nervous system development. Low cholesterol levels, both in the CNS and in the placenta, also impact allopregnanolone synthesis since the former is the substrate for the production of the neuroactive steroid allopregnanolone (Figure 5). Increased levels of Glu release have been reported in pre-term neonates who are more prone to suffer from perinatal hypoxia compared to full-term neonates (Dhillon et al., 2018). This increment in Glu can activate glutamate receptors (NMDA, AMPA, and kainate) on oligodendrocytes, making them sensitive to excitotoxic damage following excessive receptor activation and preventing the normal development and production of myelin. Elevated gestational allopregnanolone levels suppress damaging excitotoxic levels and maintain protective levels of inhibition throughout late gestation (Shaw et al., 2020). Placental allopregnanolone is vehiculized as its precursor, progesterone, to the fetal plasma and brain, thus explaining why both circulating and brain allopregnanolone levels drop after birth (Kelleher et al., 2013; Hirst et al., 2018). Thus, in pre-term neonates, there is a decrease in allopregnanolone concentration associated with hypomyelination (Shaw et al., 2015). Animal studies using guinea pig pre-term neonates (Shaw et al., 2019) show positive effects on myelination with an allopregnanolone analog acting on GABA_A_Rs. Neurosteroid replacement has been suggested as a viable therapeutic option to improve myelination in this condition, as neurosteroids may help prevent neurodevelopmental disorders associated with pre-term birth (Shaw et al., 2019). This series of studies make apparent the impact of cholesterol metabolism dysregulation on GABAergic signaling associated with the reduced availability of GABA_A_R’s positive allosteric modulator, allopregnanolone.

As for glutamate receptors, cholesterol depletion in cultured rat cerebellar granule cells profoundly reduces NMDAR responses and increases NMDAR desensitization. In contrast, cholesterol enrichment potentiates NMDAR responses (Korinek et al., 2015). However, modulation of cholesterol in these cells had no effects on the amplitude of either AMPA or kainate receptor responses. More recently, these authors demonstrated that acute cholesterol depletion of hippocampal cultures induces a significant reduction of amplitudes of both NMDAR and AMPAR eEPSCs (Korinek et al., 2020).

Hence, GABA and glutamate receptors are directly modulated by cholesterol. The prenatal period is of particular importance due to the fine regulation of this lipid during nervous system development.

## nAChR and Cholesterol

Four decades of research on the effect of cholesterol on the peripheral nAChRs found in skeletal muscle and in the electromotor synapse of electric fish have provided us with abundant information on the multiplicity of effects of this sterol on the nAChR. Cholesterol influences nAChRs at various levels of organization—ranging from the molecular to the cellular—and within multiple time windows, covering the millisecond (single-channel properties) to the minute and hour (endocytic/exocytic trafficking) time scales, during ontogenetic development and adulthood (Barrantes, 2004, 2007).

Both muscle-type and neuronal nAChRs require lipid platforms for trafficking to the plasma membrane, and cholesterol, sphingolipids, and ceramides are important players in this process (Brusés et al., 2001; Marchand et al., 2002; Baier and Barrantes, 2007; Gallegos et al., 2008; Baier et al., 2010). Disruption of these lipid platforms leads to both impaired nAChR function and diminished cell-surface expression (Baier et al., 2010; Borroni and Barrantes, 2011). Lowering cholesterol levels disrupts lipid domains, altering nAChR nanocluster topography and mobility at the cell surface, causing rapid nAChR internalization, and compensatory gain-of-function at the single-channel level of the nAChRs remaining at the plasma membrane (Borroni et al., 2007).

Cholesterol modulation of the nAChR is related to the concentration of cholesterol in the plasmalemma (Santiago et al., 2001). Chronic pharmacological cholesterol depletion using the drug mevinolin on the CHO-K1/A5 cell line (Pediconi et al., 2004) decreases cell-surface nAChRs by inhibition of receptor exocytosis and retention of the protein at the Golgi complex, while acute methyl-β-cyclodextrin mediated cholesterol depletion reduces the amount of cell-surface nAChR by accelerating receptor endocytosis (Borroni et al., 2007). In addition, changes in the distribution of nAChRs have been observed upon cholesterol changes as studied by stimulated emission depletion (STED; Kellner et al., 2007) and single-molecule localization (SMLM; Mosqueira et al., 2018, 2020) superresolution microscopies. Moreover, changes in cholesterol levels alter the translational mobility of the receptor in the plane of the plasma membrane, as measured by fluorescence recovery after photobleaching and fluorescence correlation spectroscopy (Baier et al., 2010) and SMLM (Mosqueira et al., 2018, 2020). Pharmacologically induced cholesterol depletion leads to changes in nAChR stability, topographical distribution and long-range dynamic association with other nAChR molecules, and transiently affects the channel kinetics of those receptors remaining at the surface as a compensatory mechanism for the temporary loss of cell-surface receptors (Borroni et al., 2007). Acute cholesterol loss accelerates endocytosis and even leads to re-routing of the endocytic pathway followed by the receptor (Borroni and Barrantes, 2011). These experimentally induced changes in the nAChR mimic the conditions found in clinical settings in patients chronically treated with statins.

Less explored are the effects of cholesterol modulation on neuronal nAChR, despite the fact that 20 years have passed since the neuronal nicotinic α7 nAChR was suggested to occur in lipid rafts at the surface of the somatic spines in chick ciliary ganglion sympathetic neurons (Brusés et al., 2001). More recently, the macroscopic responses of neuronal nAChR subtypes were shown to be significantly reduced as cholesterol to phospholipid ratios increased (Báez-Pagán et al., 2016). Increments in the cholesterol/phospholipid ratio produced a reduction in the generation of macroscopic currents mediated by the α7 nAChR. α7 nAChRs in cholesterol-rich liquid-ordered lipid domains (Brusés et al., 2001; Marchand et al., 2002; Oshikawa et al., 2003; Zhu et al., 2006), may constitute a non-activatable pool of nicotinic receptors, as suggested by Báez-Pagán and coworkers (Báez-Pagán et al., 2016). Long-term inhibition of cholesterol biosynthesis by the drug lovastatin differentially augments cell-surface levels of α4β2 and α7 nAChRs in neurites and soma of rat hippocampal neurons (Borroni et al., 2020). In conclusion, dysregulation of cholesterol homeostasis affects the stability, distribution and conductance of both muscle-type and neuronal nAChRs.

## nAChRs in ASD

Altered levels of nAChRs in various brain regions of autistic individuals have been described (Anand et al., 2011). In ASD patients, lower levels of expression of the α4β2 nAChRs have been reported in the cerebellum (Lee et al., 2002) and in the parietal and frontal cerebral cortex (Perry et al., 2001; Martin-Ruiz et al., 2004). In contrast, α7 nAChR subunit expression was shown to be increased in the granule cell layer, but not in the Purkinje cells or the molecular cell layer of the cerebellum. The cerebellum is a brain region implicated in social cognition (Laidi et al., 2017) and therefore cerebellar alterations may be related to eye avoidance and reduced social attention commonly observed in the pathophysiology of ASDs (Laidi et al., 2017). With regard to other brain areas, the α4 nAChR subunit expression was not modified in the thalamus, whereas β2 and α7 subunit expression was reported to be decreased in the paraventricular nucleus and nucleus reuniens of the thalamus of ASD individuals (Martin-Ruiz et al., 2004). These thalamic nuclei have reciprocal connections with corticolimbic areas implicated in the pathogenesis of ASD (Ray et al., 2005). The above data suggest that dysregulation of nicotinic cholinergic transmission may take place in the thalamus. Interestingly, studies using β2 nAChR subunit knockout mice (Granon et al., 2003), reported that β2-containing nAChRs regulate executive and social behaviors.

Sleep disruption and anxiety disorders are common in patients with ASD. Mice that express a knockout of *CHRNA4*, the gene coding for the α4 subunit (Anand and Lindstrom, 1992), are more anxious (Ross et al., 2000) whereas β2 nAChR subunit knockout animals present an abnormal sleeping pattern (Léna et al., 2004). Anand and coworkers (Anand et al., 2011) suggested that modifications in nAChR expression may compensate for an altered homeostasis of neural networks, secondary to the lack of a proper balance of excitatory and inhibitory signaling in ASD patients. Furthermore, the imbalance of excitatory and inhibitory synaptic transmission in neuronal circuits during prenatal and postnatal brain development may be responsible for the difficulties encountered in the establishment of language processing and social behavior in ASD (Rubenstein and Merzenich, 2003). Given that nAChR-mediated cholinergic transmission in the frontal cortex increases the threshold for activating glutamatergic synapses (Couey et al., 2007) and stimulates GABA release (Alkondon et al., 2000), any alteration in the expression of nAChR genes in this period will negatively impact neuronal circuits.

With regards to genetic mutations involving *CHRNA7*, the α7 nAChR gene located in the chromosome region 15q13.3, there is also evidence that may correlate to autistic-like phenotypes (Yasui et al., 2011). Interestingly, α7 nAChR *CHRNA7* null mutant mice present increments of interleukin 6 (IL6) levels in the mutant fetal brain due to maternal immune activation (Wu et al., 2015). In addition, these mice present increased behavioral deficits. Remarkably, gestational choline supplementation improved the fetal brain response to maternal immune activation and precluded some of the behavioral abnormalities observed in these offspring (Wu et al., 2015). Many studies from animal models and *in vivo* models support the idea that the stimulation of α7 nAChR has pro-cognitive effects (Marotta et al., 2020). The implication of the PI3K/Akt signaling cascade crosstalk with the Wnt/-catenin signaling cascade and both transcriptional and non-transcriptional effects of catenin have been associated with α7 nAChR (Deutsch et al., 2016). One of the best-characterized effects of valproic acid, a well-established pharmacological rat model of ASD, is the stimulation of the canonical Wnt signaling pathway (Kwan et al., 2016). Changes in canonical Wnt signaling during prenatal brain development can have a profound impact on brain function and induce autism-like features (Fang et al., 2014). *Adenomatous polyposis coli* (*APC*), a critical component of the canonical Wnt pathway, is a key player in neural plasticity, learning, and memory in mice. Knock-down of the *APC* gene displayed ASD-like behaviors in a mouse model, with concomitant increases in synaptic spine density, elevated frequency of miniature excitatory postsynaptic potentials, and enhanced LTP (Zhou et al., 2007). We demonstrated that Wnt 7 induced presynaptic colocalization of α7 nAChR and APC in mature hippocampal rat neurons (Farías et al., 2007), raising the possibility that enhancement of the Wnt signaling-pathway during prenatal brain development may also dynamically modulate neurotransmitter release by altering α7- nAChRs levels at synaptic terminals. Adequate cholesterol levels are also needed for proper Wnt signaling to occur. Recent studies have shown that defects in cholesterol synthesis reduce Wnt signaling, causing abnormalities in craniofacial development and neural crest cell differentiation (Sezgin et al., 2017; Castro et al., 2020). Similarly, inadequate cholesterol modulation of presynaptic nAChR may produce alterations in the timing for KCC2 expression in prenatal brain and delay or even impede synaptic pruning, thus having a role in the observed increased dendritic arborization, in addition to the synaptic density modifications due to reduced APC expression.

Substantial reduction in nAChR α4 subunit expression in the piriform cortex was reported in a pentylenetetrazol (PTZ)-kindled mice model. The piriform cortex is thought to be involved in the development of seizures in several forms of epilepsy. It is located between the limbic and cortical networks, allowing it to facilitate epileptogenesis and propagate epileptic activity (Pollock et al., 2014). If we take into consideration that epilepsy is associated with several psychiatric disorders, including ASD, it is not surprising that these authors found an interaction between autism-like neurobehavioral deficits and nAChRs. Moreover, they studied the therapeutic effects of a nicotinic agonist, ABT-418, a nootropic and neuroprotective drug that exhibits high affinity for α4β2 nAChR that has been used in AD and attention deficit hyperactivity disorder, and found statistically significant improvements in some ASD psychiatric symptoms (Takechi et al., 2016).

To sum up, α7 and α4β2 nAChR subtypes have become possible therapeutic targets in ASD. Good results have been obtained so far with the α7 agonist galantamine in a randomized, double-blind, placebo-controlled trial. Galantamine treatment showed a statistical improvement in irritability, lethargy, and social withdrawal with good tolerability (Ghaleiha et al., 2014). Donepezil, another drug used in psychiatric disorders including AD, increases the availability of acetylcholine at the synapses, thus enhancing cholinergic transmission. The drug has also shown good safety and tolerability profile and demonstrated improvement in behavioral dysfunctions (Hardan and Handen, 2002). Finally, 3-(2,4-dimethoxybenzylidene)-anabaseine (DMXB-A), a selective partial agonist of α7 nAChRs, has shown efficacy in a randomized, double-blind crossover trial on neurocognitive improvements in subjects with schizophrenia spectrum disorders (Olincy et al., 2006) with similar effects in two adult patients with ASD (Olincy et al., 2016).

## Concluding Remarks

Within the last decade studies on cholesterol metabolism in the brain have gained momentum because of the implications in neurodegenerative diseases. A full understanding of the brain’s cholesterol metabolism and its role in various CNS diseases, including ASD, will require further investigation. Since neurons and glia undergo major changes during development, any alterations of membrane properties due to dysregulation of cholesterol homeostasis will negatively impact the various pathways linked to sterol processing, leading to a great variety of pathophysiological outcomes. Gain and loss of function of neuronal receptors inevitably affect the excitatory-to-inhibitory synaptic equilibrium and synaptic plasticity. We have illustrated the multiple modulatory effects exerted by cholesterol on nAChR function, and how the latter affects in turn glutamatergic and GABAergic synaptic transmission. The prenatal period is particularly vulnerable because of the key events requiring precise timing and synchronization that occur during early neurodevelopmental stages. Clinically oriented research will surely address these critical events to identify possible therapeutic targets.

## Author Contributions

FB and AV conceived the work, searched the literature, and wrote the manuscript. Both authors contributed to the article and approved the submitted version.

## Conflict of Interest

The authors declare that the research was conducted in the absence of any commercial or financial relationships that could be construed as a potential conflict of interest.

## Publisher’s Note

All claims expressed in this article are solely those of the authors and do not necessarily represent those of their affiliated organizations, or those of the publisher, the editors and the reviewers. Any product that may be evaluated in this article, or claim that may be made by its manufacturer, is not guaranteed or endorsed by the publisher.
